# Developmental defects and impaired network excitability in a cerebral organoid model of KCNJ11 p.V59M-related neonatal diabetes

**DOI:** 10.1038/s41598-021-00939-7

**Published:** 2021-11-03

**Authors:** Gokhan Dalgin, Andrew K. Tryba, Ashley P. Cohen, Soo-Young Park, Louis H. Philipson, Siri Atma W. Greeley, Alfredo J. Garcia

**Affiliations:** 1 Section of Endocrinology, Diabetes and Metabolism, Departments of Medicine and Pediatrics, Kovler Diabetes Center, The University of Chicago, Chicago, IL USA; 2 Section of Pediatric Neurology, Department of Pediatrics, The University of Chicago, Chicago, IL USA; 3 Chicago Medical School, Rosalind Franklin University, North Chicago, IL USA; 4 Section of Emergency Medicine, Department of Medicine, Institute for Integrative Physiology, Grossman Institute for Neuroscience, The University of Chicago, Chicago, USA

**Keywords:** Stem cells, Pluripotent stem cells, Induced pluripotent stem cells

## Abstract

The gene *KCNJ11* encodes Kir6.2 a major subunit of the ATP-sensitive potassium channel (K_ATP_) expressed in both the pancreas and brain. Heterozygous gain of function mutations in *KCNJ11* can cause neonatal diabetes mellitus (NDM). In addition, many patients exhibit neurological defects ranging from modest learning disorders to severe cognitive dysfunction and seizures. However, it remains unclear to what extent these neurological deficits are due to direct brain-specific activity of mutant K_ATP_. We have generated cerebral organoids derived from human induced pluripotent stem cells (hiPSCs) possessing the *KCNJ11* mutation p.Val59Met (V59M) and from non-pathogenic/normal hiPSCs (i.e., control/WT). Control cerebral organoids developed neural networks that could generate stable synchronized bursting neuronal activity whereas those derived from V59M cerebral organoids showed reduced synchronization. Histocytochemical studies revealed a marked reduction in neurons localized to upper cortical layer-like structures in V59M cerebral organoids suggesting dysfunction in the development of cortical neuronal network. Examination of temporal transcriptional profiles of neural stem cell markers revealed an extended window of SOX2 expression in V59M cerebral organoids. Continuous treatment of V59M cerebral organoids with the K_ATP_ blocker tolbutamide partially rescued the neurodevelopmental differences. Our study demonstrates the utility of human cerebral organoids as an investigative platform for studying the effects of KCNJ11 mutations on neurophysiological outcome.

## Introduction

Neonatal diabetes mellitus is a monogenic disorder of which about 30% are caused by dominant heterozygous mutations in *KCNJ11*^1^. These mutations commonly cause sustained activation of the K_ATP_ channel that in turn, produces a sustained hyperpolarization within cells. These gain-of-function mutations in the K_ATP_ channel cause hyperglycemia due to failed insulin secretion from pancreatic beta cells^2^. K_ATP_ channels are expressed not only in the pancreas but also in other organs such as brain and muscle, where the activating mutation may have a direct contribution to tissue specific dysfunction observed in patients with neonatal diabetes. For example, patients with mutations in *KCNJ11* suffer from neurological deficits, subtle to severe learning disorders and cognitive disorders such as autism spectrum-like disorder^3,4^, which correlate with the degree of channel dysfunction.

K_ATP_ channels play an important role in coupling neuronal metabolism to electrical excitability and neurotransmitter release^5^. These activity-dependent processes influence the development of neuronal circuits during neurogenesis^6^. For example, in mouse corticogenesis, the generation of neural subtypes derived from neural stem cells (NSC) can be directed by manipulating membrane potential^7^. Membrane potential can also alter the balance between different channel conductances that play a role in the normal maturation of hippocampal neurons and the local neural circuit^8^. Advances in human induced pluripotent stem cell and 3D cell culture technology have made it possible to generate brain tissue called cerebral organoids, in vitro^9,10^. Cerebral organoid technology provides a good model for understanding neural differentiation as well as the effects of genetic variation on gene expression of brain development and disease^11–13^. In this study, we generated cerebral organoids from human induced pluripotent stem cells (hiPSCs) carrying either a non-pathogenic/control or KCNJ11 V59M mutant allele, to compare the effects of this gain of function K_ATP_ mutation on maturation of the cortical network activity. Our data suggest that in V59M organoids, the NSC fails to differentiate and migrate normally. This decreased neurogenesis results in defective neural circuit formation and activity. Pharmacological treatment of mutant organoids with the K_ATP_ channel blocker tolbutamide partially rescues the molecular defects caused by hyperpolarization of the cell membrane. This study provides the first direct evidence that a mutant KCNJ11 channel causes neurological deficits in patient hiPSC derived brain tissue.

## Results

### Generation of cerebral organoids from control and V59M mutant iPSC lines

We generated induced pluripotent stem cells (iPSCs) from a female patient^14^ with permanent neonatal diabetes and neurodevelopmental delay without known epilepsy due to KCNJ11 V59M to study the effects of hyperactive KCNJ11 channel activity on brain function (Fig. S1, panels A–D). The reprogramed control and V59M iPSCs showed normal morphology (Fig. S1A,C) and had a normal 46;XX karyotype by standard metaphase spreads and G-banded karyotyping (Fig. S1, panels B and D). Sanger sequencing confirmed that the presence of the mutant channel allele was sustained after reprograming (Fig. S1, panels E, F). Both iPSC lines expressed pluripotent markers OCT4 and TRA-1–60 (Fig. S1, panels G, H) and OCT4 and SOX2 (Fig. S1, panels I, J) and pluripotency was demonstrated by direct differentiation into three embryonic germ layers (Fig. S1, panels K-P). Both iPSC lines differentiated into ectoderm (PAX6; Fig. S1, panels K, L), mesoderm (BRA; Fig. S1, panels M, N) and endoderm (SOX17; Fig. S1, panels O, P).

**Figure 1:**
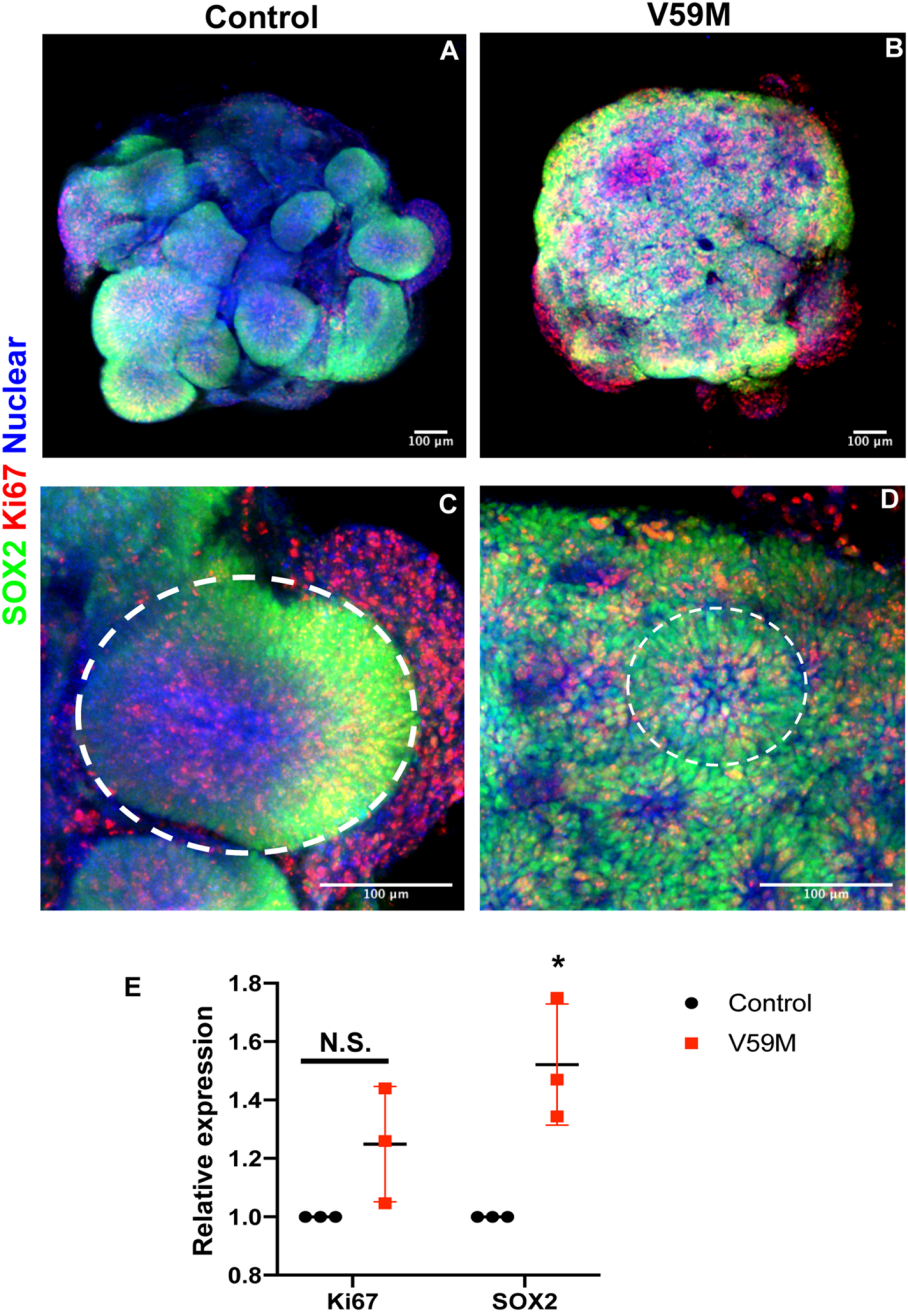
Representative confocal images (merged z-stacks) of 15-day-old control **(A,C)** and V59M mutant **(B,D)** cerebral organoids. Whole mount immunolabeling for SOX2 (green), Ki67 (red) with nuclear staining TO-PRO-3 (blue). Scale bar 100 µm. Dashed circles; representatives of ventricle-like structures and neural rosettes in control **(C)** and V59M mutant **(D)** cerebral organoids respectively. **(E)** Relative levels of *Ki67* and *SOX2* by real-time qPCR. N.S., not significant. *P < *0.05; t-test,* two tailed distribution.

We successfully generated cerebral organoids from two clones for both control and V59M lines, with one clone per each line selected for detailed studies described below. Both lines generated uniform round embryoid bodies (EB) with smooth edges and diameters of about 500 µM (Fig. S2A). EBs then developed to form optically translucent edges demonstrating neural induction (Fig. S2A). During the next 10 days, EBs started to show neuroepithelial bud expansion (Fig. S2A) that then showed fluid-filled cavities reminiscent of ventricles (Fig. S2A, panels C and D). Tissues continued to grow and mature until they formed cerebral-like structures (Fig. S2A).

### V59M mutant organoids show impaired neurodevelopment

Younger cerebral organoids (~ 15 days old) exhibit multiple large and continuous neuroepithelium-like structures similar to a ventricle (Lancaster et al., 2013). To compare the timing of formation of these structures between control and V59M organoids, we performed immunofluorescence staining for markers of neural progenitors (SOX2) and proliferating cells (Ki67). Control organoids showed multiple large and continuous neuroepithelia surrounded by a ventricle (from minimum 6 independent experiment with minimum of 24 organoids) (Fig. S2, panels B-D). By contrast, V59M organoids formed mostly small neural rosette-like neuroepithelia (from minimum 6 independent experiment with minimum of 18 organoids) (Fig. S2, panels E–G). These data suggest that the V59M organoids may have an impaired development. However, previous studies have shown that cerebral organoids morphological and molecular inter- and intra-variability^15^ may explain, at least in part, the formation of this phenotype. To address these issues, we performed immunofluorescence staining for SOX2 and Ki67 on cleared whole organoids. Analysis of control and V59M organoids from 3 independent experiments with minimum of 18 organoids per group suggested that large and continuous neuroepithelium was consistent across the control organoids (n = 18) (Fig. 1A,C) whereas, V59M organoids uniformly produced neural rosette-like structures (n = 14) (Fig. 1B,D). These data suggest that the developmental phenotype that is observed in V59M mutant organoids is uniform within the experimental groups.

**Figure 2:**
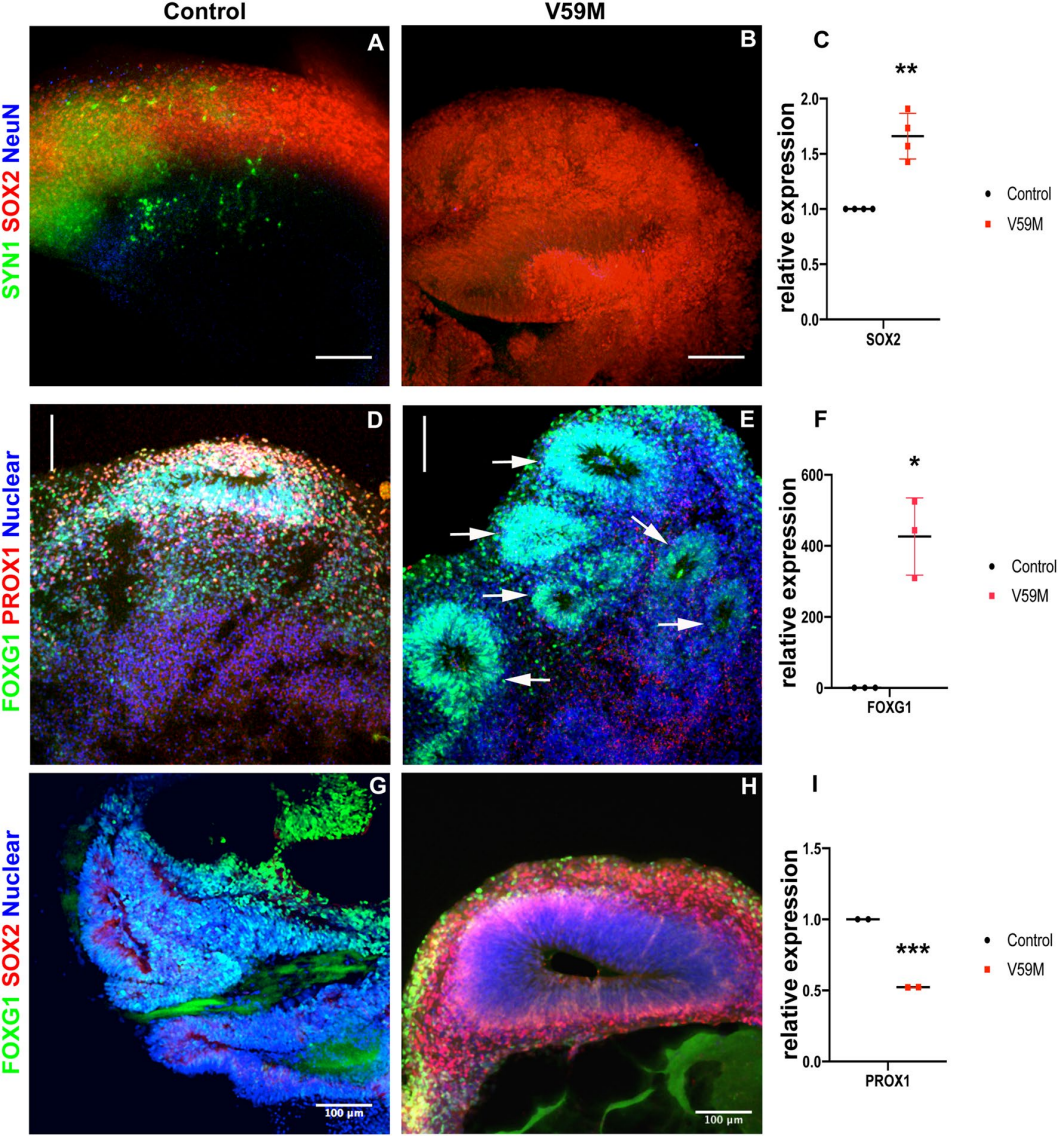
Representative confocal images (merged z-stacks) of 45–50-day-old control **(A,D,G)** and V59M mutant **(B,E,H)** cerebral organoids. Immunofluorescence for SOX2 (Red, **A,B,G,H**), SYN1 (green, **A,B**), FOXG1 (**D,E,G,H**), PROX1 (**D,E**) with nuclear staining TO-PRO-3 (blue). Neural rosettes (arrows). Scale bar 100 µm. Relative levels of *SOX2* **(C)**, FOXG1 **(F)** and PROX1 **(I)** by real-time qPCR. *P < 0.05, **P < 0.008, ***P < *0.0005; t-test,* two tailed distribution.

We also performed qRT-PCR analysis to quantify neuronal progenitor gene expression in V59M organoids relative to control organoids. The qRT-PCR results showed that expression of *SOX2* was higher in V59M mutant organoids. There was no significant difference in the expression of *Ki67* (Fig. 1E). The increase in *SOX2* expression in mutant organoids is sustained at later stages of cerebral organoid development (Fig. 2C). These data suggest that the neurodevelopment phenotype in V59M organoids might be a consequence of sustained expression of *SOX2* in neural progenitors impairing differentiation to more mature neuronal stages.

### Aberrant expression of brain regionalization and specification markers in V59M mutant organoid

To examine the differentiation further, we performed immunofluorescence staining for a presynaptic marker synapsin 1 (SYN1), neuronal progenitor marker SOX2, and a mature neuron marker NeuN. In 45 day cleared whole organoids, there was strong expression of SYN1 and scattered NeuN expressing cells in controls, whereas expression of these two genes was reduced in V59M mutants (Fig. 2A,B). Both control and V59M organoids expressed the neural progenitor marker SOX2, although a thicker layer of cells expressing SOX2 (Fig. 2A,B) was observed in mutant organoids. Consistent with this elevated SOX2 protein expression V59M mutant organoids showed higher expression of *SOX2* mRNA detected by qRT-PCR analysis (Fig. 2C). Thus, sustained expression of SOX2 in V59M organoids may impair neurogenesis.

Next we examined the expression of brain regionalization markers for early forebrain and hippocampus by immunostaining for FOXG1 and PROX1 respectively. We detected expression of both FOXG1 and PROX1 in both 50-day control and V59M organoids (Fig. 2D,E). However, the V59M organoids showed an increase in FOXG1 staining and a decreased in PROX1 staining whereas the staining for these markers was similar in the control organoids. We confirmed these results by calculating the total fluorescence for FOXG1 and PROX1 in control and V59M mutant organoids (Fig S3). Analysis of total fluorescence intensity showed that FOXG1 immunofluorescence was ~ 2.3 times higher in V59M mutant organoids than controls (Fig. S3A-C) and PROX1 immunofluorescence showed ~ 2.24 times less fluorescence intensity in V59M mutant organoids when compared to controls (Fig. S3D-F). Furthermore, quantitative RT-PCR showed higher levels of FOXG1 mRNA and lower levels of PROX1 mRNA in V59M organoids compared to controls (Fig. 2F,I). In addition, the V59M organoids continued to have numerous neural rosette-like structures expressing FOXG1 (Fig. 2E arrows). We hypothesized that the sustained expression of SOX2 could be associated with the observed defects and analyzed the expression FOXG1 and SOX2 by immunostaining. Control organoids showed no overlap between cells expressing FOXG1 and SOX2 (Fig. 2G). By contrast, V59M mutant organoids had cells co-expressing FOXG1 and SOX2 (Fig. 2H) suggesting that the FOXG1^+^/SOX2^+^ cells failed to progress towards more differentiated cell types.

### Impaired neural differentiation in V59M mutant organoids

**Figure 3:**
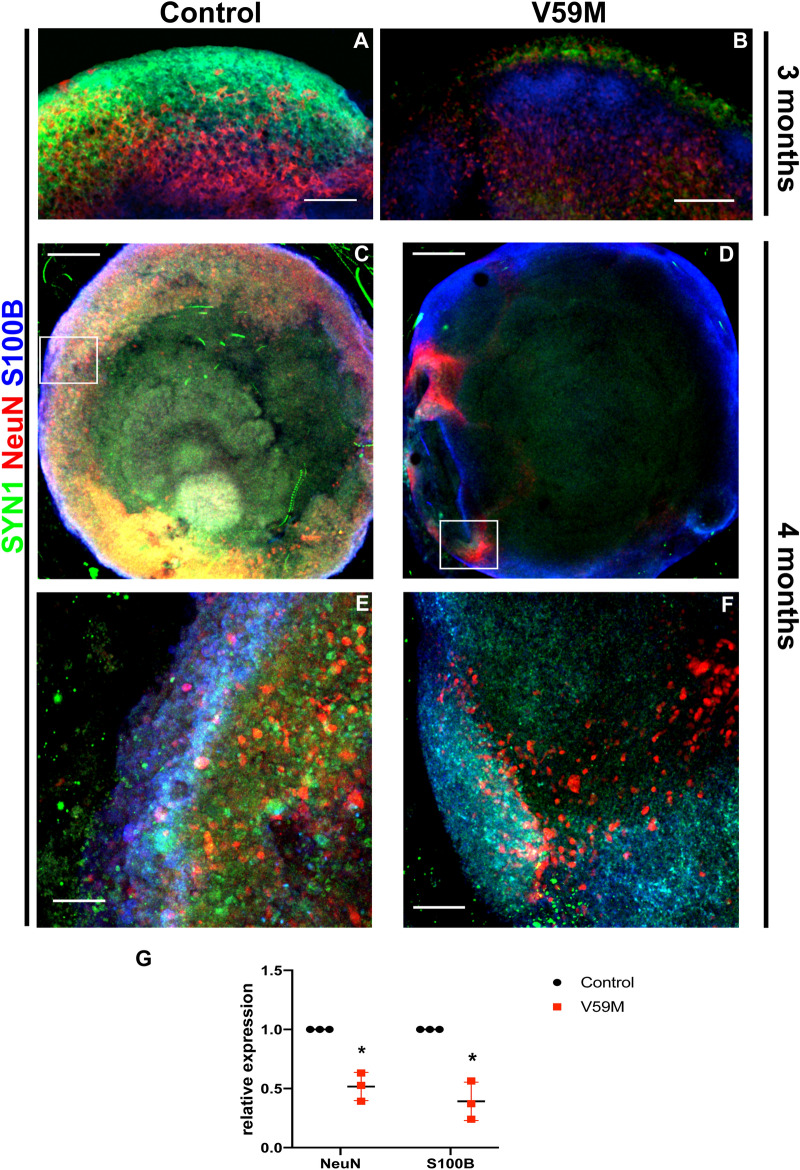
Mutant organoids have a defective neural network. Representative confocal images (merged z-stacks) of control and V59M mutant organoids at 3 months **(A,B)** and at 4 months **(C–F)**. Immunofluorescence for SYN1 (green), NeuN (red) and S100B (blue). (**E,F)** are high power images of the boxed area in (**C,D)** respectively. Scale bars 100 µm **(A,B,E,F)** and 500 µM **(C,D)**. **(G)** Relative levels of *NeuN* and *S100B* by real-time qPCR. *P < *0.003; t-test,* two tailed distribution.

To investigate the differentiation state of the organoids in more detail, we stained the organoids for the presynaptic marker synapsin 1 (SYN1), mature neuron marker NeuN and astrocyte marker S100B in 3-month old organoids. Control organoids exhibited layers of cells expressing SYN1, NeuN and S100B whereas V59M organoids appeared to have decreased expression of these markers (Fig. 3A,B). We confirmed these results using 4-month cleared whole control and V59M organoids. Whole organoid immunostaining showed that control organoids established a deep and continuous neural network expressing layers of SYN1, NeuN and S100B (Fig. 3C,E). The V59M organoids lacked continuous cell layers expressing SYN1 and NeuN indicated no or a poorly organized neural network (Fig. 3D,F). Consistent with these results, the V59M mutant organoids had decreased expression of *S100B* and *NeuN* mRNA (Fig. 3G).

### Neural network activity in control and V59M cerebral organoids

Despite differences in the expression of neuronal and synaptic markers, no spontaneous neuronal network activity was observed in either control (n = 6) or V59M mutant (n = 6) cerebral organoids at 3 mM extracellular KCl (Fig. S4A). By raising extracellular KCl, a minority of cerebral organoids from control (n = 1 of 6) and mutant (n = 2 of 6) cerebral organoids exhibited spontaneous bursting (Fig. S4B) yet the proportion of spontaneous bursting cerebral organoids were similar between groups (Fig. S4C). These data suggested that majority of neuronal networks from both groups were functionally immature despite the expression differences in neuronal and synaptic markers at day 45. Thus, to resolve whether the differences in expression of neuronal and synaptic markers between control and mutant cerebral organoids reflected differences in network activity, we allowed the cerebral organoids to develop further and assayed network activity at an older age range.

**Figure 4:**
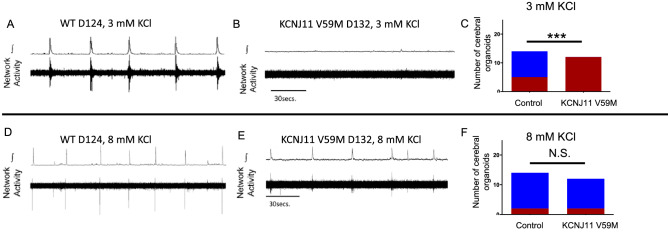
(**A–C)** Between days 73–183, none of the V59M mutant CO display network bursting in ACSF containing 3 mM KCl while approximately ~ 60% of WT CO generate bursting activity. (**D–F)** However, increasing ACSF KCl to 8 mM, increases the number of both WT and mutant V59M CO that generate bursting to about ~ 80%. *** P < 0.0007, *N.S.* not significant. Fishers exact test. Blue = bursting and Red = not bursting.

**Figure 5:**
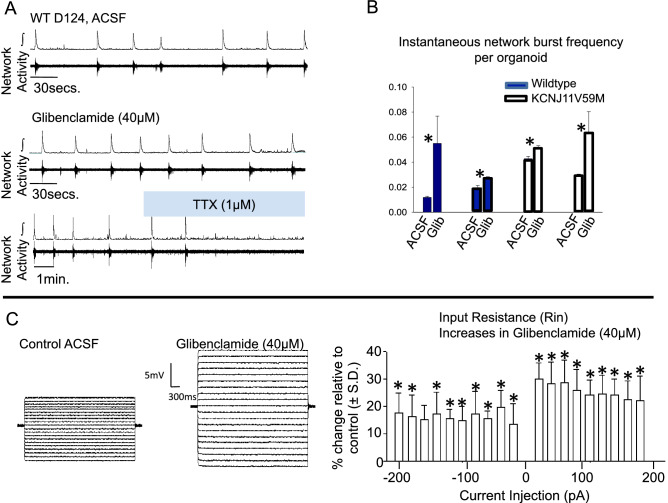
**(A)** Representative traces of cerebral organoid (CO) network activity in ACSF (top), glibenclamide (Glib, middle) and Tetrodotoxin (TTX). Spontaneous network activity is increased by Glib, and eliminated following bath-application of TTX, a voltage-sensitive sodium channel blocker. (**B**) Paired comparisons instantaneous burst frequency of individual control (WT, n = 2) and mutant (KCNJ11 V59M, n = 2) organoids. (**C**) Blocking KATP channels with glibenclamide increases input resistance.

In older organoids, the majority of control cerebral organoid networks (n = 9 of 14; mean age 145.57 ± 28.74 days) displayed neuronal network activity in 3 mM extracellular KCl (Fig. 4A); whereas, no mutant V59M cerebral organoids (n = 0 of 12, mean age 152.0 ± 38.19 days) generated network bursting activity (Fig. 4B). Thus, the proportion of spontaneously bursting cerebral organoids in 3 mM KCl was greater in control versus the V59M mutant cerebral organoids (Fig. 4C). However, elevating extracellular KCl eliminated the difference in spontaneous network activity as the majority of networks from normal (n = 12 of 14) and mutant cerebral organoids (n = 10 of 12) generated synchronized network bursting (Fig. 4D–F). Furthermore, extracellular recordings of demonstrated that the KATP channel blocker, glibenclamide (40 µM), consistently increased spontaneous network activity (Fig. 5A,B, n = 4). Intracellular recordings also revealed that glibenclamide increased input resistance among individual cells (Fig. 5C, n =  4). These data demonstrated a functional role of KATP channels in neuronal networks of both normal and mutant cerebral organoids.

### V59M organoids have defects in laminar organization of neocortex development

**Figure 6:**
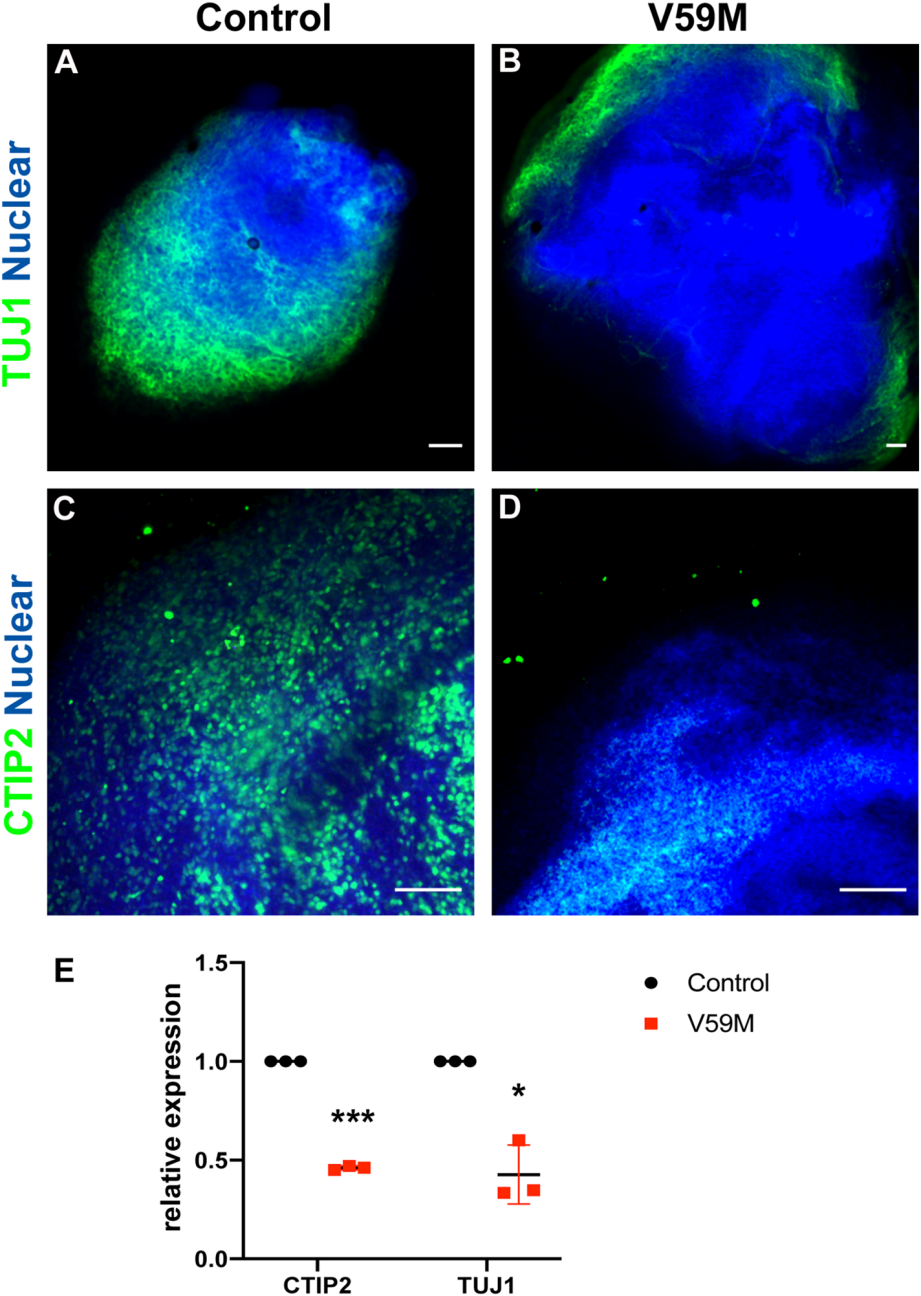
Representative confocal images (merged z-stacks) of control **(A–C)** and V59M **(B–D)** mutant organoids at 4 months. Immunofluorescence for TUJ1 **(A,B,** green**)**, CTIP2 **(C,D,** green**)** with nuclear staining TO-PRO-3 (blue). Scale bar 100 µm **(A,B)**, 200 µm **(C,D)**. **(E)** Relative levels of *CTIP2* and *TUJ1* by real-time qPCR. *P < 0.003, ***P < *0.0001; t-test,* two tailed distribution.

**Figure 7:**
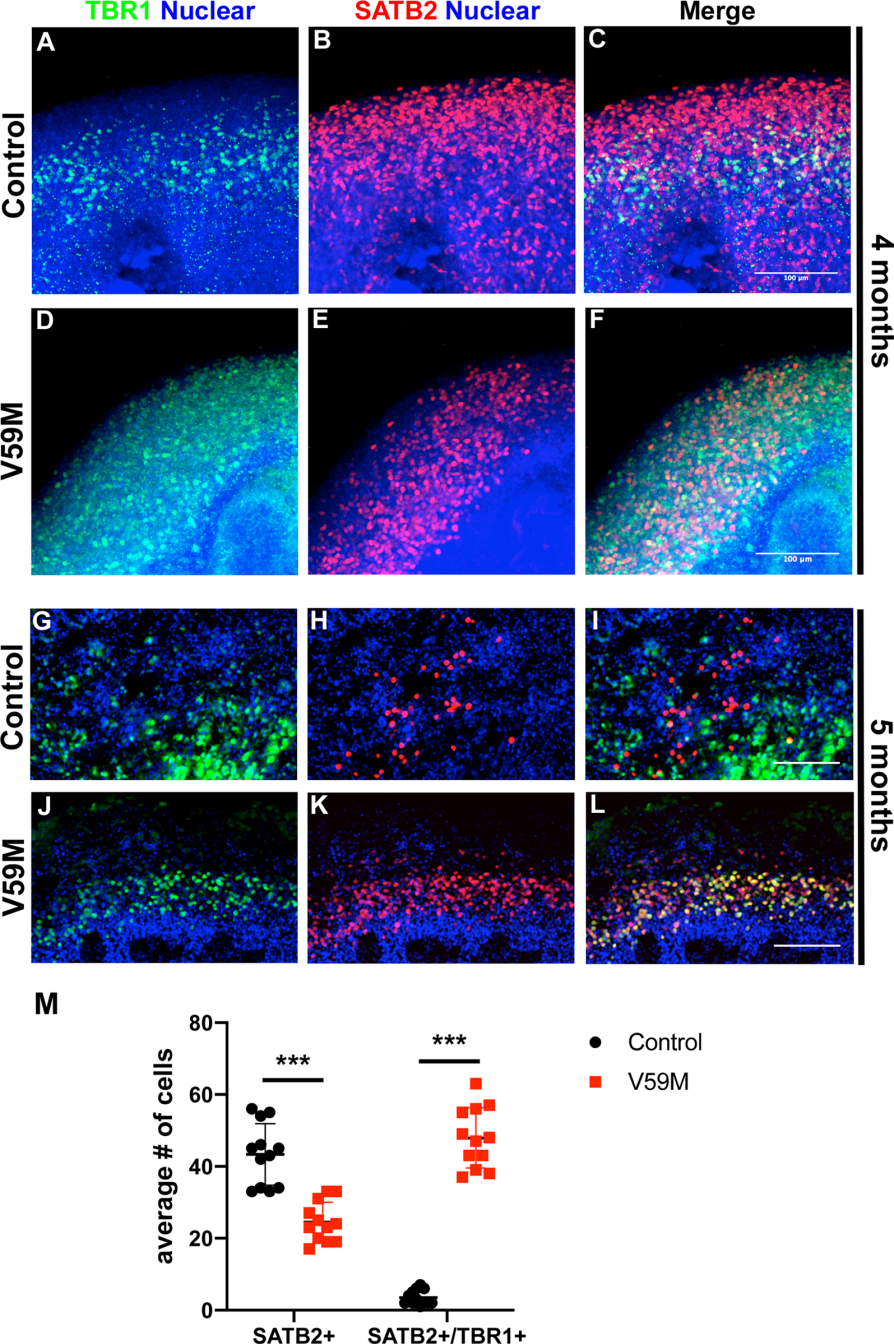
Representative confocal images (merged z-stacks) of control **(A–C,G–I)** and V59M mutant **(D–F,J–L)** organoids at 4 months **(A–F)** and at 5 months **(G–L)**. Immunofluorescence for TBR1 (green), SATB2 (red) with nuclear staining TO-PRO-3 (blue). Scale bar 100 µm **(A–F)** and 200 µM **(G–L)**. **(M)** Average numbers of SATB2 + and SATB2 + /TBR1 + cells. ***P < *0.00001; t-test,* two tailed distribution.

Our molecular and electrophysiological analysis revealed that V59M mutant organoids have defects in neural maturation and network activity. We showed that V59M mutant organoids could produce neural progenitors and have decreased neurogenesis. Therefore, we predicted that neural progenitors fail to generate the proper number of neuronal subtypes typical of the cerebral cortex. To test this hypothesis, we compared expression of a neuron specific marker TUJ1 and deep layer cortical marker CTIP2. Immunofluorescence analysis of 4-month old cleared control and V59M mutant whole organoids showed that both TUJ1 and CTIP2 are uniformly expressed in control organoids (Fig. 6A,C). By contrast, expressions of these markers were significantly decreased in V59M mutant organoids (Fig. 6B,D). Consistent with these results V59M organoids had a decrease in the expression of *TUJ1* and *CTIP2* (Fig. 6E). These data suggested that the decrease in neurogenesis might be due to defects in generation of neuronal subtypes in V59M mutant organoids. Therefore, we performed immunofluorescence analysis to compare the expression of preplate/deep layer neuron marker TBR1 and subcortical projection neuron marker SATB2 between control and V59M mutant organoids. At 4 months, both control and V59M mutant organoids expressed TBR1 and SATB2 in a pattern similar to developing neocortex (Fig. 7A–F). In the control organoids there were distinct expression patterns of TBR1 and SATB2 where TBR1 mostly expressed in lower layers and SATB2 in upper layers (n = 9 from 2 independent experiments, Fig. 7A–C). Interestingly, in V59M mutant organoids expressions of TBR1 and SATB2 were unlike in control organoids and mostly co-expressed both markers (n = 11 from 2 independent experiments, Fig. 7D–F). This co-expression pattern of TBR1 and SATB2 resembled a developing young embryonic neocortex; therefore, we compared the expression of these markers in slightly older control and V59M mutant cerebral organoids. In 5-month old control organoids cells expressing TBR1 and SATB2 were clearly separated and SATB2 expressing cells were exclusively seen in upper layers (avg. # of SATB2^+^ cells = 43.33 ± 8.6 and avg. # of SATB2^+^/TBR1^+^ cells = 3.5 ± 2.02, Fig. 7G–I,M). Interestingly cells solely expressing TBR1 and SATB2 were largely lacking in V59M mutant organoids instead lower layer cells continued to co-express TBR1 and SATB2 (avg. # of SATB2^+^ cells = 24.5 ± 5.5, and avg # of SATB2^+^/TBR1^+^ cells 47.91 ± 8.38 Fig. 7J–L,M). These data indicated that in V59M mutant organoids proper laminar organization of the neocortex appears to be disrupted.

### Pharmacological treatment to rescue neurodevelopmental defects in V59M mutants

**Figure 8:**
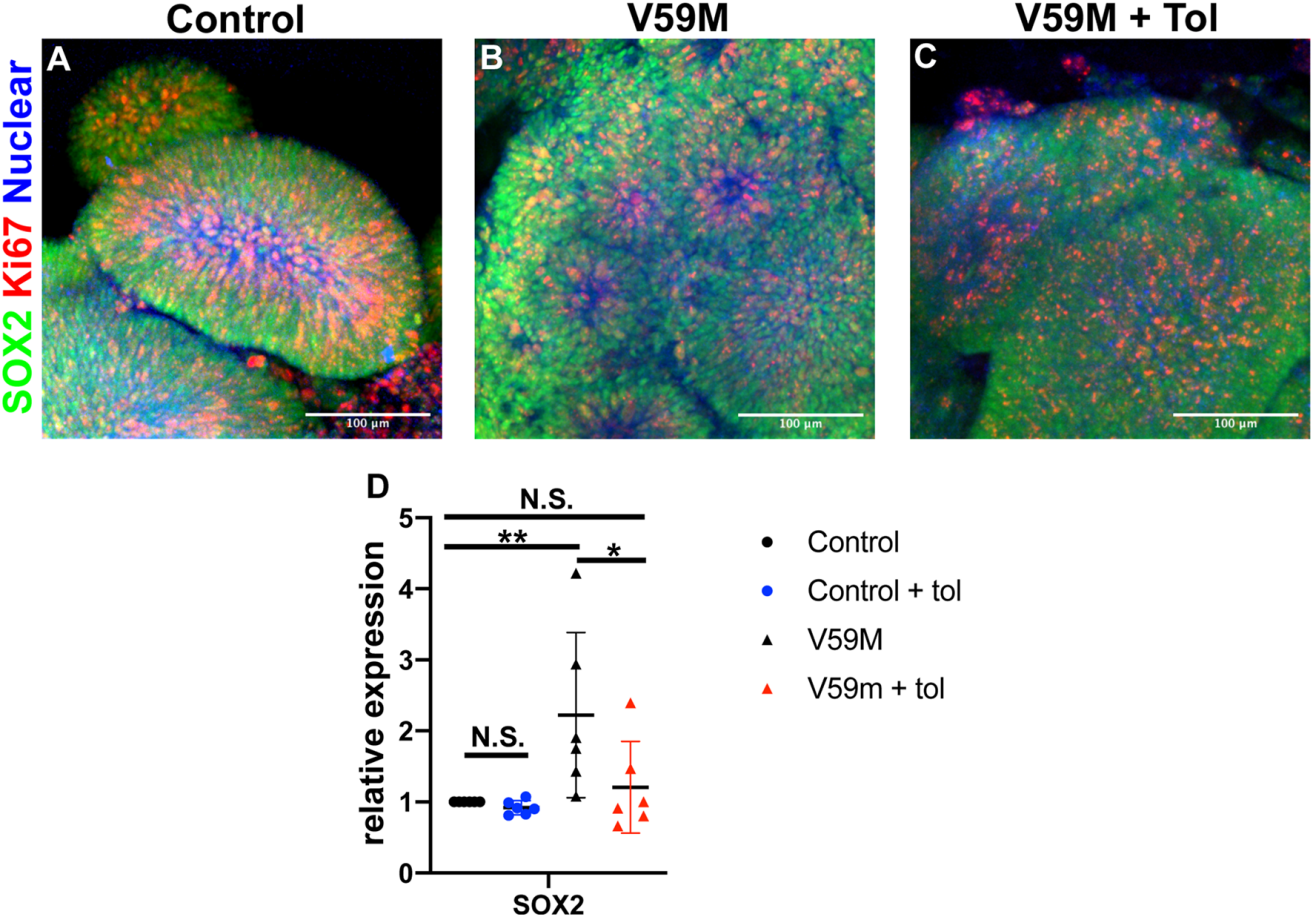
Pharmacological treatment of cerebral organoids. Representative confocal images (merged z-stacks) of control **(A)** V59M mutant **(B)** and V59M + Tol **(C)** organoids at day 15. Immunofluorescence for SOX2 (green), Ki67 (red) with nuclear staining TO-PRO-3 (blue). Scale bar 100 µm. Relative levels of *SOX2* by real-time qPCR. N.S. not significant, *P < 0.05, **P < *0.01*; one way ANOVA, multiple comparisons with Bonferroni correction.

Sulfonylureas (SU) are a class of drugs that are effective in treating diabetes patients with activating mutations in *KCNJ11*; furthermore, SU treatment also correlates with some improvement in neurodevelopmental outcomes^16,17^. Therefore, we tested whether treatment with a first-generation potassium channel blocker, Tolbutamide (Tol) could reduce the neurodevelopmental defects observed in V59M mutant organoids. At day 10 of organoid maturation, a subset of control and V59M mutant organoids media were supplemented with 200 µM of Tol and refreshed every 2 days until analysis. We performed whole mount immunofluorescence staining for markers of neural progenitors SOX2 and proliferating cells Ki67 to determine whether Tol treatment would revert V59M mutant neural rosette-like neuroepithelia to a neuroepithelium that are observed in control organoids. On day 15, neuroepithelium in Tol treated and untreated control organoids were indistinguishable from each other (Fig. 8A and data not shown). However, V59M mutant organoids treated with Tol showed neuroepithelium structures similar to control specimens when compared to untreated V59M organoids (Fig. 8B,C). Consistent with these results qRT-PCR analysis between d15 to d50 showed that the increase in *SOX2* transcripts in V59M mutant organoids was significantly decreased with Tol treatment (Fig. 8D). These data suggest that Tol treatment may partially rescue neurodevelopmental defects. Altogether, our data revealed that the neurodevelopmental defects that are observed in *KCNJ11* V59M mutant patients can be modeled by cerebral organoids using hiPSCs. Furthermore, this platform can be used to find novel drug therapies that are much needed by these patients.

## Discussion

Recently advancements in personalized medicine platforms using stem cell derived tissues make it possible to find novel drug therapies for individuals suffering from genetic disorders^18,19^. Our patient iPSC-derived cerebral organoid platform made it possible to uncover pathologies related to mutation in a neonatal diabetes gene *KCNJ11*. By performing analysis in whole organoids and across different organoid batches, our experimental design accounted for variations among organoids of the same genetic background. However, as the organoids generated are not from isogenic controls, we cannot discount the potential contribution that differences in genetic background may play in our results. We coupled quantitative mRNA expression with immunohistochemistry studies, yet differences in gene expression may not always reflect differences in protein expression. Future studies will need to further determine the extent of transcriptional differences are reflected in protein levels between control and mutant organoids. Despite these limitations the cerebral organoid platform has provided important insights into the potential consequences of the genetic mutation in *KCNJ11* can cause during human neurodevelopment.

The KCNJ11 gene encodes an ATP-sensitive potassium (K_ATP_) channels that provide a critical link between neuron metabolic activity and changes in neuron electrical activity as well as neurotransmitter release^20^. Mutations in KCNJ11 can cause diabetes and neurocognitive dysfunction^2,3,21^. By using human stem cell-derived cerebral organoids we find that the KCNJ11 mutation (V59M) disrupts organization and impairs maturation of cortical neural networks. This is in part due to V59M organoids forming and maintaining small neural rosette-like neuroepithelia unable promote proper neural differentiation. Treatment of V59M mutant cerebral organoids with Tolbutamide (Tol) caused SOX2 expression to return to levels similar to that in control organoids. These data are consistent with the clinical observation that neurodevelopmental disabilities may be improved by early therapeutic SU intervention in KCNJ11 mutant patients^22^. Therefore, we predict that SU treatment could also rescue the expression of mature neural markers, yet this possibility remains to be tested.

Our experiments demonstrate that prolonged expression of neural stem/progenitor cell (NSC) marker SOX2 in V59M mutant organoids might reduce neuronal progenitor differentiation (Figs. 1, 2, 3). As brain development is a complex process that requires synchronization of many signaling cascades that are controlled by spatiotemporal activity of transcription factors, the prolonged expression of SOX2 in mutant organoids coincided with alterations in the molecular signature of constituents within the organoid. This observation is consistent with findings in animals that constitutive expression of SOX2 has been shown to inhibit the differentiation of neural progenitor cells into neurons and results in the maintenance of progenitor attributes^23^. Therefore, increased Sox2 expression could indicate prolonged lingering of progenitor identities that could contribute to the delay in maturation.

In addition to differences in SOX2, V59M mutant organoids showed an excessive increase FOXG1 expression (Fig. 2). *FOXG1* is a transcription factor important to normal forebrain development. In mice, loss of the gene results in microcephaly^24^. Loss of function analysis also reveals that *FOXG1* plays an important role regulating NSC proliferation and suppressing premature neuronal differentiation^24,25^. By contrast, overexpression of FOXG1 with the downstream factor TLE1 maintains neural stem cells in proliferative state^26^. Thus, we propose that the prolonged expression of SOX2 and FOXG1 maintain cells in a neural precursor state and causes an aberrant expression of FOXG1^+^/SOX2^+^ cells (Fig. 2G,H) which keep the V59M mutant organoids in a premature state.

In agreement with this study, V59M mutant organoids showed a marked reduction in PROX1 expression. *Prospero-related homeobox 1* (*Prox1*) gene is a critical regulator of embryonic and adult neurogenesis. During embryonic development PROX1 promotes NSC cycle exit and neural differentiation while also inhibiting gliogenesis^27^. Loss of *PROX1* in mice diminished the integration of GABAergic interneurons into superficial layers^28^. Consistent with the role of *PROX1* in interneuron development and generation of superficial layers we found that deep-layer neurons (i.e., CTIP2^+^) and superficial-layer neurons (i.e., SATB2^+^) were poorly localized in mutant cerebral organoids (Fig. 7). This improper localization of these cell types to the upper cortical layers was reflected in the decreased expression of SYN1 and NeuN (Fig. 3) in the V59M mutant cerebral organoid. While the interior of the organoids may not receive sufficient nutrients and become necrotic and/or hypoxic, these conditions apply to both control and V59M mutant organoids. It is possible that hypoxia may exacerbate differences to mutant and the control organoids. Nonetheless, our results strongly suggest that the V59M mutation dysregulates neural circuit formation.

Treating mutant cerebral organoids with Tol , restored SOX2 levels similar to that found in controls. This effect of Tol was also co-incidental with a reversion from the neural rosette structure to neural epithelium-like morphology found in the control organoid. These findings suggest that the aberrant hyperpolarizing activity of KCNJ11 in the V59M mutant is major factor in neural development and circuit formation. Depolarization inhibits the proliferation of radial glial cells (NSC)^29,30^ and promotes NSC differentiation^31,32^. Whereas, neural progenitor hyperpolarization regulates sequential generation of neuronal subtypes^7^. Thus, our findings are consistent with these previous reports that implicate the importance of bioelectric activity to the regulation of progenitor cell properties and suggests that the gain of function mutation caused by V59M may promote a state unfavorable to cell-cycle exit. Our data suggest that increased expression in progenitor marker SOX2 and non-significant increase in the expression of proliferation marker Ki67 (Fig. 1) may provide the unfavorable state to cell-cycle exit.

While the mutant KCNJ11 channel activity impairs neural progenitor development, our electrophysiological studies show that mutant cerebral organoids are capable of forming functional neuronal networks whose excitability is suppressed under basal conditions. However, by elevating extracellular potassium levels the difference between the number of spontaneously active control and mutant cerebral organoids is eliminated.

In conclusion, our work has provided the first evidence that the mutant KCNJ11 channel activity dysregulates circuit formation and network excitability in human samples. Using the cerebral organoid platform, we disentangled the confounding effects associated with neonatal diabetes from the direct influence of the V59M mutation on neural precursors and neurons. Thus, we have demonstrated that organoids provide greater resolution into the mechanisms underlying cellular and neurophysiological phenomena comorbid with complex metabolic disease.

## Materials and methods

### Study approval

In accordance with National Institute of Health guidelines, all experiments using human iPSCs were performed with the approval of The University of Chicago Institutional Review Board (#16935B) and Institutional Biosafety Committee. An informed consent was obtained from all subjects and parental and/or legal guardian.

### Derivation and maintenance of iPSCs

A chiPSC1.5 iPSC cell line was derived from normal female adult human dermal fibroblast (Lonza CC-2511). Briefly, an early passage (P2) was plated at 3500-cells/cm^2^ in FGM-2 fibroblast growth medium-2 BulletKit (Lonza, CC-3132). Reprograming of human fibroblasts was achieved by using ReproRNA-OKSGM a single-stranded RNA replicon vector that contains the five reprograming factors: OCT4, KLF-4, SOX2, GLIS1 and c-MYC (Stem Cell Technologies (STC) 05930). The manufacturer’s protocol was followed for reprograming the human fibroblasts. Peripheral blood mononuclear cells (PBMCs) were isolated from peripheral blood a female patient (Støy et al., 2008) carrying the KCNJ11 mutation V59M. PBMCs were reprogramed by the Cincinnati Children’s Hospital Pluripotent stem cell facility. Following adaptation to mTeSR1 and matrigel conditions, cell lines were cryopreserved and tested for mycoplasma (PromoKine PK-CA20-700–20). Standard metaphase spreads and G-banded karyotypes were determined by Wi-Cell Laboratories (Madison, WI).

### Cerebral organoid culture conditions and maintenance

We generated cerebral organoids following protocols published by Lancaster et al., 2014^33^ or by using STEMdiff Cerebral Organoid Kit (STC #08570) with modifications. We did not observe any morphological or molecular differences between these two methods. All culture media were prepared based on manufacturer’s recommendations; iPSCs were dissociated when cells were at 80% confluent using GCDR (SCT # 07174). To form embryoid bodies (EBs), dissociated cells were suspended in EB formation medium containing 10 µM Y-27632 and seeded at 9000-cells/well densities in a 96-well ULA plate (Corning 7007). On day 7 after neural induction, EBs were kept on 96-well ULA plate with media containing 2% Matrigel (Corning #354277). On day 10, EBs were transferred onto 6-well plates (Greinier-Bio-One 657185 or Corning 3471). From this point forward, organoids were kept in maturation medium on an orbital shaker (Innova 2000 75 rpm or Fisher-88861021 105 rpm) and media replaced every other day. Between days 20–30, maturation medium was switched to BrainPhys (SCT 05790) media supplemented with SM1 (SCT 05711), N2A (SCT 07152), NEAA (Gibco 11140050), Glutamax (Gibco 35050061), Insulin (Sigma I9278), BME (EMD-Millipore 805740) and 2 ng/ml BDNF (R&D 248-BDB-010/CF). Media was replaced with fresh media every 3 days. For experiments with sulfonylurea treatment, culture media is supplemented with 200 µM Tolbutamide (Sigma T0891) and refreshed every other day.

### Immunofluorescence, tissue clearing and imaging

Organoids were fixed in 4% paraformaldehyde for 20 min at room temperature followed by washing in PBS three times for 15 min each. Tissues were incubated in 30% sucrose in 1× PBS overnight at 4 ^o^C and then embedded in OCT (Fisher 23–730-571) and cryosectioned at 20–30 µm. For whole organoid immunofluorescence, tissues were fixed in 4% paraformaldehyde for 20 min at room temperature. To wash out PFA, organoids were kept in PBS on an orbital shaker overnight at room temperature. The next day, PBS was replaced with a permeabilization solution (2% Triton X-100 (Sigma T8787), in PBS solution containing 0.05% sodium azide (Fisher S2271-25)). Organoids were kept on an orbital shaker at room temperature for minimum of 2–3 days depending on the size of the organoids. At the end of the permeabilization, organoids were incubated in blocking buffer (10% normal donkey serum-Gemini Bio 100–151, 1% Triton X-100 in PBS solution containing 0.2% sodium azide), on an orbital shaker at 4^o^ C overnight. Organoids were incubated with primary antibodies in antibody dilution buffer (1% normal donkey serum 0.2% Triton X-100 in PBS solution containing 0.2% sodium azide) on an orbital shaker at 4^o^ C for 2 days in 1:400 dilutions of the following primary antibodies: CTIP2 (EMD Millipore MABE1045), FOXG1 (Abcam ab18259), Ki67 (BD Biosciences 550609), NeuN (EMD Millipore MAB377), PROX1 (EMD Millipore MAB5654), S100B (Abcam ab41548), SATB2 (Abcam ab51502), SOX2 (Abcam ab5603), SYN1 (Synaptic Systems 106011C2), TBR1 (Abcam ab31940), TUJ1/Anti-beta-tubulin III (Sigma T2200). Organoids were washed in washing buffer (3% NaCl and 0.2% Triton-X 100 in PBS at 4 ^o^C overnight). The organoids were then incubated with secondary antibodies in antibody dilution buffer on an orbital shaker at 4^o^ C for 1 day with 1:1000 dilutions of the following secondary antibodies: anti-goat DyLight 488 (Thermo SA5-10086), anti-mouse DyLight 488 (Thermo SA5-10166), anti-rabbit DyLight 488 (Thermo SA5-10038), anti-rat DyLight 488 (Thermo SA5-10026), anti-mouse DyLight 550 (Thermo SA5-10167), anti-rat DyLight 550 (Thermo SA5-10027, anti-mouse DyLight 650 (Thermo SA5-10169). The next day, organoids were counterstained with the nuclear marker TO-PRO-3 (Invitrogen S33025) and then washed in washing buffer overnight at 4 °C on an orbital shaker. Organoids were then transferred to RapiClear (SUNJin Lab #RC147001 or #RC149001) and tissues were kept in the solution until they were cleared (usually 4–10 days depending on the size of the organoid). Sectioned or whole organoids were imaged using a Nikon C1 confocal microscope with 4×, 10× or 20× objectives and analyzed using ImageJ software. Total fluorescence was measured from two control and two V59M mutant organoids. Eight sections (confocal z-stacks) from Control organoids and six sections from V59M organoids were analyzed by using ImageJ software. Total corrected fluorescence (TCF) was measured per section by TCF = integrated density – (area of selection × mean fluorescence of background readings).

### Quantitative real-time PCR (qRT-PCR)

Total RNA was isolated from 4 to 6 organoids per group using a Qiagen RNAeasy mini kit (Qiagen catalog # 74104), according to manufacturer’s instruction. The cDNA was prepared using iScript cDNA synthesis kit (Bio-Rad 170-8891). The sequences of TaqMan primers used in this study are listed in Table 1. cDNA was amplified using StepOnePlus Real-Time PCR system (Applied Biosystems 4367659). Relative expression of each sample was determined after normalization to 18S or RPL37a levels using the efficiency-corrected delta Ct method^34^ and displayed relative to an arbitrary value.

**Table 1:** Taqman Gene Expression Assays for RT-PCR.

Assay ID	Gene symbol
Hs99999901_s1	18s
Hs01102345_m1	RPL37A
Hs01102259_m1	CTIP2/BCL11B
Hs01850784_s1	FOXG1
Hs01032443_m1	MKI67
Hs00896293_m1	PROX1
Hs01370653_m1	RBFOX3/NEUN
Hs00902901_m1	S100B
Hs01053049_s1	SOX2
Hs00801390_s1	TUJ1/TUBB3

### Electrophysiology recording

Cerebral organoids were submerged in a Warner Instrument Corp. (Hamden, CT; model RC-27LD) recording chamber (~ 6 mL) under circulating oxygenated artificial cerebrospinal fluid (ACSF, flow rate 17 ml/min, total circulating volume = 200 mL). The ACSF contained in mM: 118 NaCl, 3 KCl, 1.5 CaCl_2_, 1 MgCl_2_, 25 NaHCO_3_, 1 NaH_2_PO_4_ and 30 D-glucose, equilibrated with carbogen (95% O_2_ and 5% CO_2_, pH  7.4). All chemicals were obtained from Sigma (St. Louis, MO, U.S.A.). As temperature can alter neural activity and network behavior^35,36^, bath temperature was monitored and maintained at 30  ± 0.7 °C using a Warner Instrument model TC-344B temperature regulator with a Warner Instrument inline solution heater (SH-27B) and Warner Instrument bath heating platform (PH-1); bath temperature at various locations within the bath was uniform.

### Electrophysiology neural network population recordings

Extracellular recordings were obtained with glass suction electrodes positioned on the surface of the cerebral organoids. The extracellular signals were amplified 10,000-fold and filtered between 0.25 and 1.5 kHz using a pre-amplifier (built by JFIE Electronics at The University of Chicago, Chicago, IL, USA) and a Model P-55 A.C. amplifier (Grass Instruments Technologies, Astro-Med, Inc., West Warwick RI, USA). The multi-unit population activity was rectified and integrated using a custom-made electronic integrator with a time constant of 70 ms (JFIE Electronics). Both the raw and integrated population activity data were digitized with a Digidata acquisition system (Molecular Devices, CA), stored in a Microsoft compatible computer and analyzed off-line.

Control recordings were made over a period of 10 min in ACSF containing 3 mM extracellular potassium chloride concentration, [KCl]. The [KCl] was elevated to achieve bursting by raising in a stepwise fashion from 3 to 5 mM then to 8 mM for 10 min before subsequently commencing recording network activity (10 min). Antagonists were bath applied to circulating ACSF at the following concentrations: TTX (Alomone labs T-550) was bath applied at 1 µM. Glibenclamide (Tocris 0911), an antagonist of the K_ATP_ channel was bath-applied at 40 µM.

### Intracellular patch-clamp recordings

Intracellular whole-cell current-clamp recordings from cerebral organoids were obtained with a MultiClamp 700B amplifier (Molecular Devices). Patch electrodes were manufactured from filamented borosilicate glass tubes (Clark G150F-4; Warner Instruments) and filled with an intracellular solution containing (in mm): 140 K-gluconic acid, 1 CaCl_2_, 10 EGTA, 2 MgCl_2_, 4 Na_2_ATP and 10 mM HEPES. Experiments were then performed in the whole-cell current clamp mode. Membrane voltage (V_m_) values were corrected for the liquid junction potential as calculated using pClamp 10 software (Molecular Devices).

### Input resistance changes

Constant current (I) injection steps were used to evoke changes in membrane potential (V_m_) and these values were used to calculate input resistance (R_in_) according to Ohm’s law (V_m_ = I*R_in_). Significant changes in R_in_ after bath applying the K_ATP_ channel antagonist glibenclamide were assessed using Student’s one-sided t-test. A Fisher’s exact test was performed to compare the differences in the proportion of bursting to no bursting networks in normal and mutant cerebral organoids.

## Supplementary Information

Supplementary Figure S1.

Supplementary Figure S2.

Supplementary Figure S3.

Supplementary Figure S4.

Supplementary Figure Legends.
