# Supplementary figures and images for: Developmental defects and impaired network excitability in a cerebral organoid model of KCNJ11 p.V59M-related neonatal diabetes
Source: Sci Rep. 2021 Nov 3;11:21590. doi: 10.1038/s41598-021-00939-7 (PMC8566525; doi:10.1038/s41598-021-00939-7)

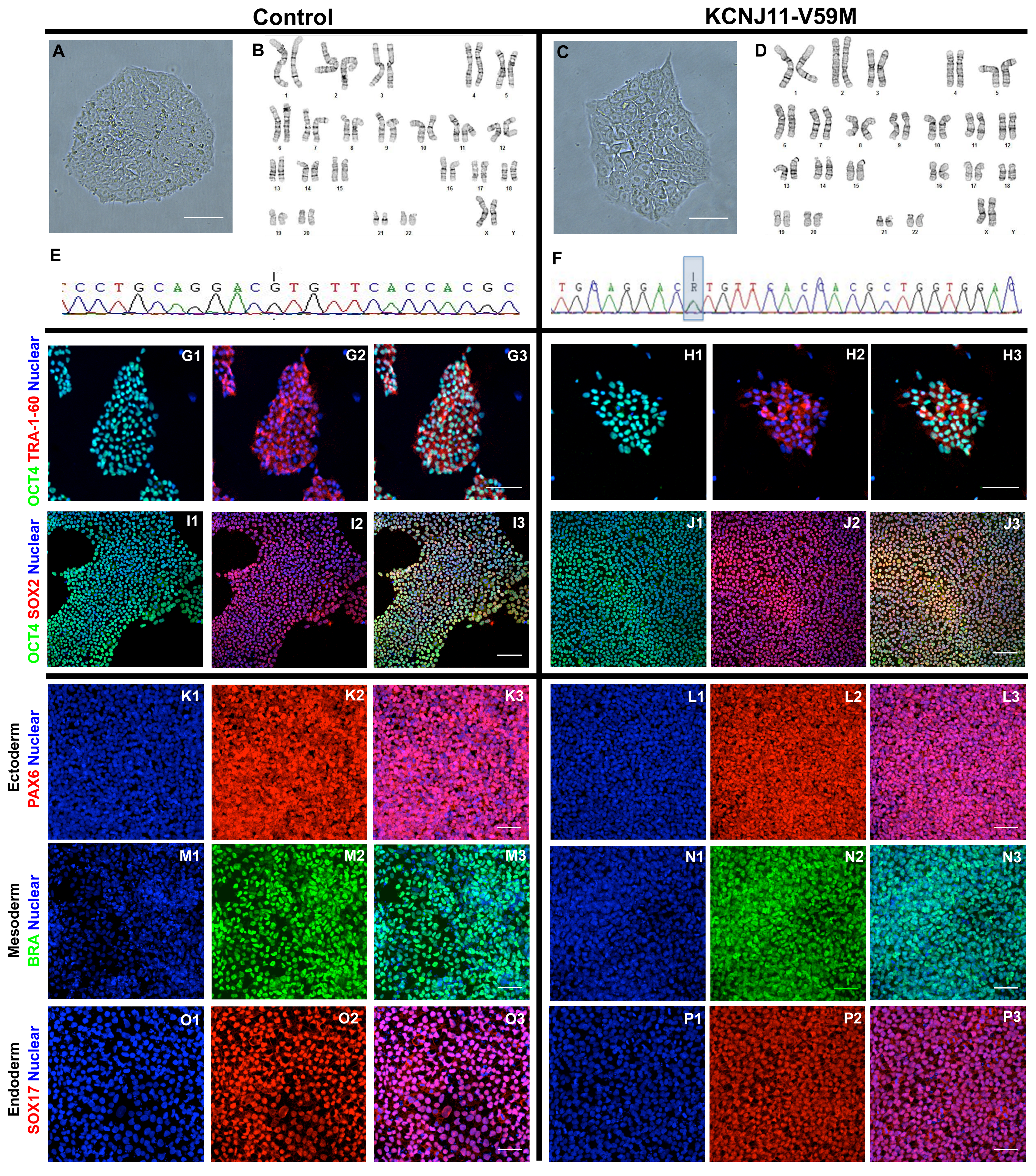

Supplement: Supplementary file 1 — Supplementary Figure S1. [file 41598_2021_939_MOESM1_ESM.tif]

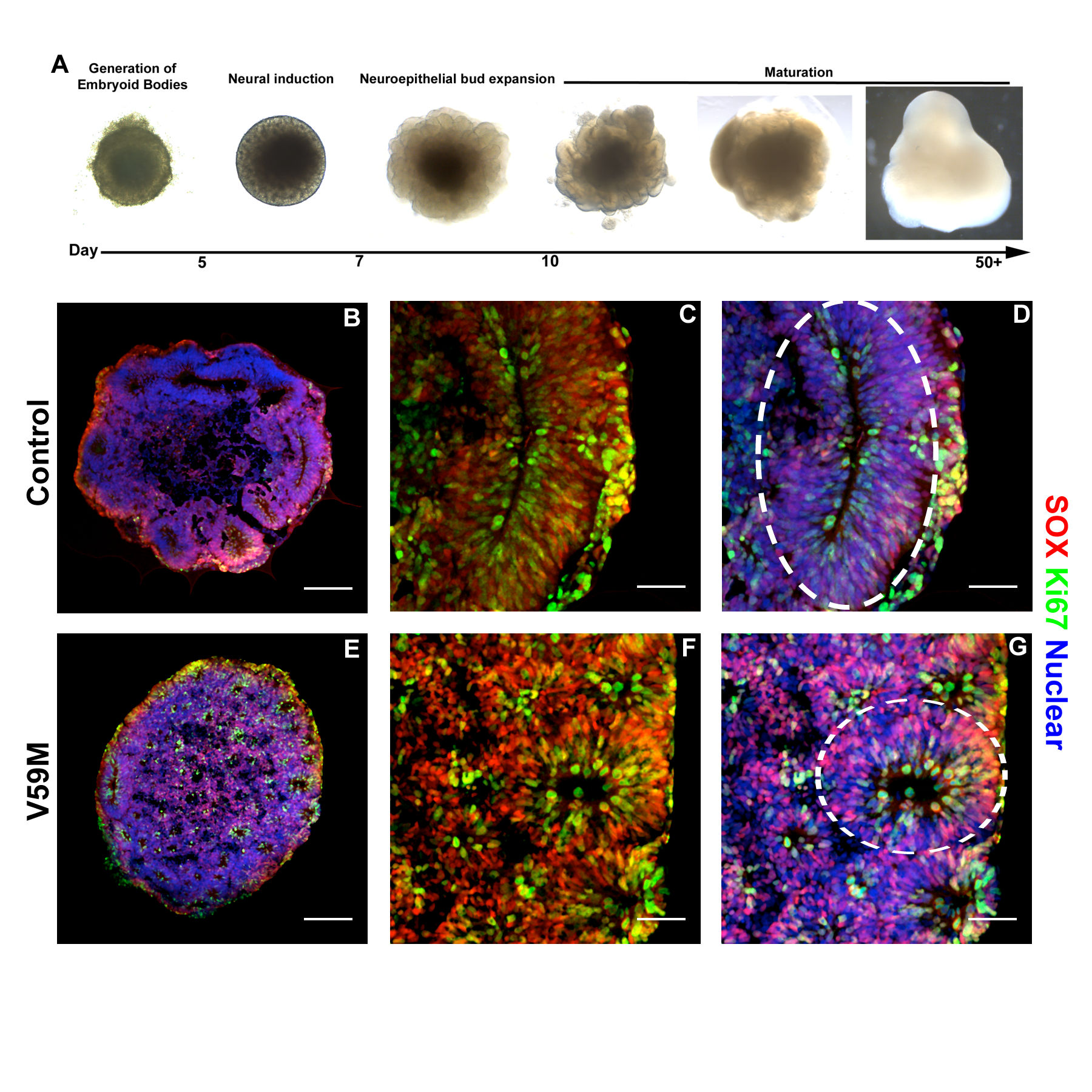

Supplement: Supplementary file 2 — Supplementary Figure S2. [file 41598_2021_939_MOESM2_ESM.tif]

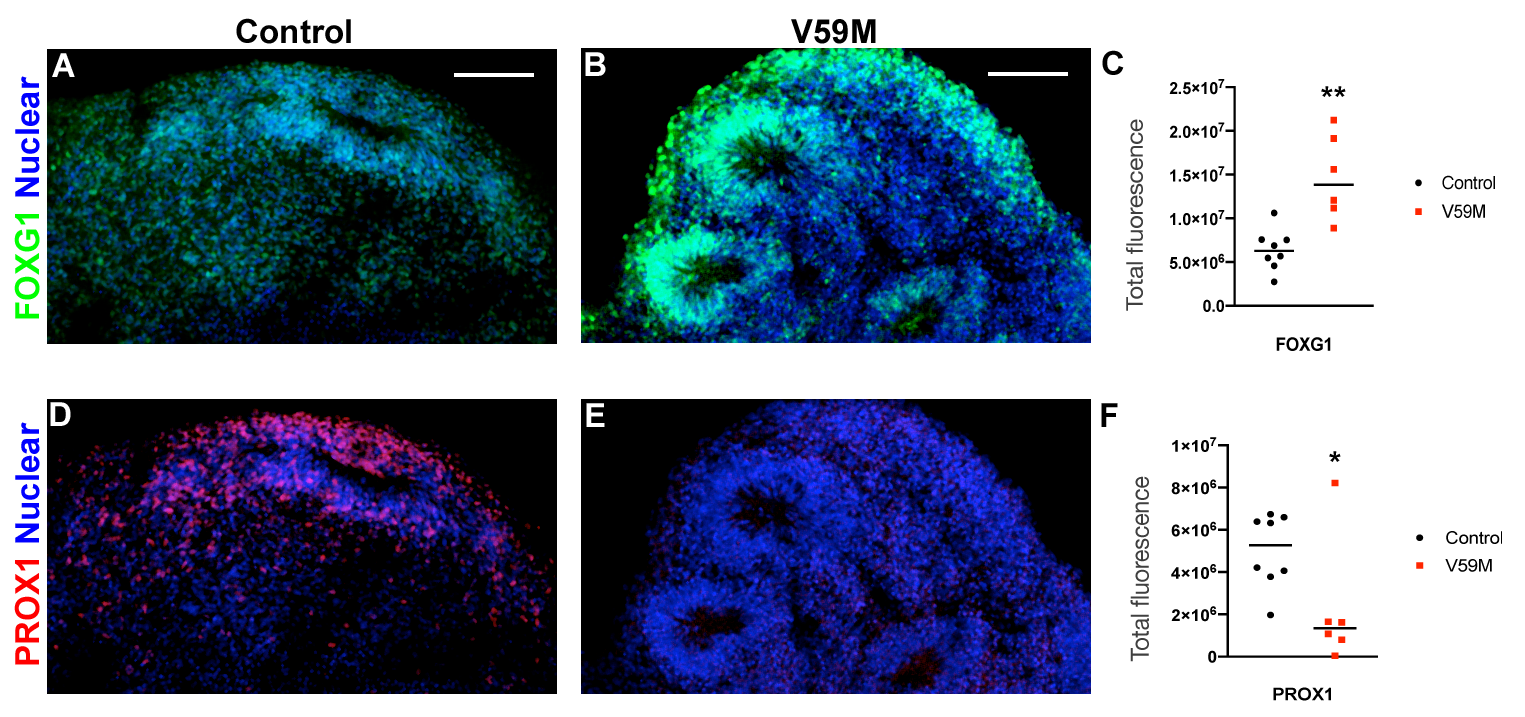

Supplement: Supplementary file 3 — Supplementary Figure S3. [file 41598_2021_939_MOESM3_ESM.tif]

A

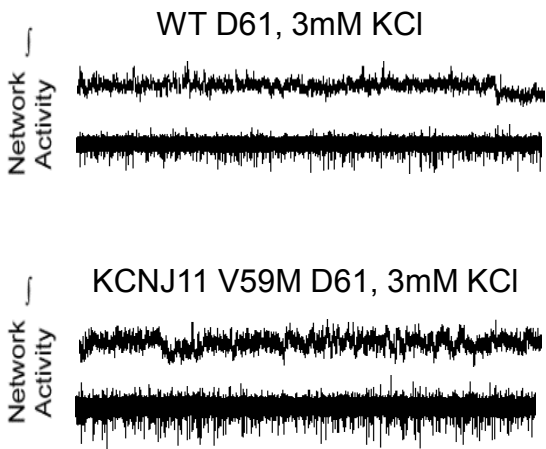

B

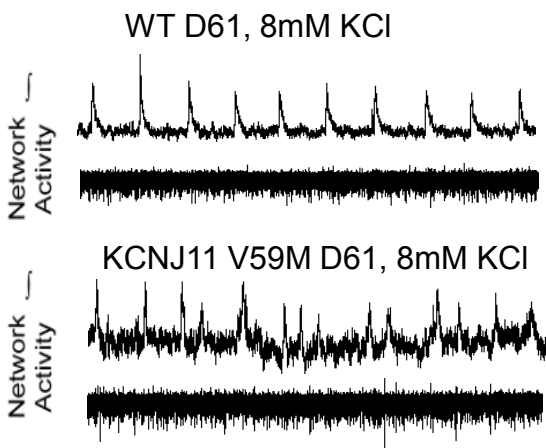

C

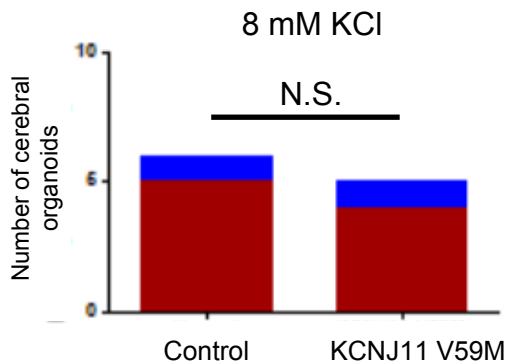

Supplement: Supplementary file 4 — Supplementary Figure S4. [file 41598_2021_939_MOESM4_ESM.pdf]
