# Supplementary material for: Developmental defects and impaired network excitability in a cerebral organoid model of KCNJ11 p.V59M-related neonatal diabetes
Source: Sci Rep. 2021 Nov 3;11:21590. doi: 10.1038/s41598-021-00939-7 (PMC8566525; doi:10.1038/s41598-021-00939-7)
Supplement: Supplementary file 5 — Supplementary Figure Legends. [file 41598_2021_939_MOESM5_ESM.docx]

**Supplemental Figures**

**Supp Figure 1.** Generation and characterization of iPSC lines. Bright field images **(A)** control and **(C)** V59M ipsc colonies showed normal morphology, scale bars 50 µm. **(B, D)** Karyotype analysis by Giemsa-banding. Sanger sequencing for control **(E)** and V59M **(F)** iPSC lines. Confocal images (merged z-stacks) of iPSC. Immunofluorescence staining for pluripotency markers OCT4 (green), TRA-1-60 (red) **(G-H)** and OCT4 (green), SOX2 (red) with nuclear staining TO-PRO-3 (blue) **(I-J),** scale bar 100 µm. *In vitro* differentiation analysis for the control **(K, M, O)** and the V59M **(L, N, P)** iPSC lines. Confocal images (merged z-stacks) of ectoderm **(K-L)**, mesoderm **(M-N)** and endodermal **(O-P)** lineages analyzed by immunofluorescence staining for PAX6 (red), BRA (green) and SOX17 (red) respectively, nuclear staining TO-PRO-3 (blue), scale bars 50 µm.

**Supp Figure 2. (A)** Key stages of cerebral organoid development from iPSC. Confocal images (merged z-stacks) of 15-day-old control **(B-D)** and V59M mutant **(E-G)** cerebral organoids. Immunofluorescence staining for SOX2 (red), Ki67 (green) with nuclear staining TO-PRO-3 (blue). Dashed circles; representatives of ventricle-like structures and neural rosettes in control **(D)** and V59M **(G)** mutant cerebral organoids respectively. Scale bars 100µm.

**Supp Figure 3.** Total fluorescence measurements for FOXG1 and PROX1 immunofluorescence from control and V59M organoids. Confocal images (merged z-stacks) of 46 day old control **(A, D)** and V59M **(B, E)** cerebral organoids. Total fluorescence for 8 different sections from 2 control organoids and 6 different sections from 2 V59M mutant organoids were plotted to present the differences for FOXG1 **(C)** and PROX1 **(F)**. Scale bars 100µm. **P<0.05, **P<0.002; t-test,* two tailed distribution.

**Supp Figure 4. (A)** Between days 45-61 neither WT or V59M mutant CO display network bursting in ACSF containing 3 mM KCl. **(B-C)** However, about ~20% of both WT and V59M mutant CO at days 45-61 generated network bursting when ACSF [K^+^]_o_ is elevated to 8 mM. N.S. not significant. Blue = bursting and Red = not bursting.
